# Effects of HCV Clearance with Direct-Acting Antivirals (DAAs) on Liver Stiffness, Liver Fibrosis Stage and Metabolic/Cellular Parameters

**DOI:** 10.3390/v16030371

**Published:** 2024-02-27

**Authors:** Joana Ferreira, Manuel Bicho, Fátima Serejo

**Affiliations:** 1 Institute for Scientific Research Bento Rocha Cabral, 1250-047 Lisbon, Portugal; manuelbicho@medicina.ulisboa.pt; 2 TERRA, ISAMB, Genetics Laboratory, Lisbon Medical School, University of Lisbon, 1649-028 Lisbon, Portugal; fatima_serejo@netcabo.pt; 3 Gastroenterology and Hepatology Department, Hospital de Santa Maria, 1649-028 Lisbon, Portugal

**Keywords:** chronic hepatitis C, fibrosis, metabolic profile, direct-acting antivirals

## Abstract

Introduction: Chronic hepatitis C (CHC) is a clinical and pathological syndrome with various causes and is characterized by varying degrees of hepatocellular necrosis and inflammation. It is a significant cause of liver transplantation and liver-related death worldwide. The hepatic manifestations of CHC are typically characterized by slowly progressing liver fibrosis, which is a non-specific and often disproportionate response to tissue damage. A large majority of HCV patients have extrahepatic manifestations with varying degrees of severity. HCV infection is a risk factor for cardiovascular disease and diabetes mellitus, which increases insulin resistance, oxidative stress, and iron overload and causes chronic systemic inflammation. HCV infection is treated using direct-acting antivirals (DAAs) with cure rates of over 95 percent, minimal side effects, and shorter therapeutic courses. Despite the effective elimination of the virus, it seemed pertinent to understand to what extent HCV clearance eliminates or attenuates all the systemic alterations already induced by the virus during infection and chronicity. Objectives: Our study aimed to determine whether eliminating HCV with DAAs alters the severity of liver disease (liver stiffness and liver fibrosis stage by TE) and the metabolic/cellular profile of patients with CHC. Materials and methods: A group of 329 CHC patients from a Gastroenterology and Hepatology outpatient department were prospectively studied. Of these, 134 were also studied with DAAs. The liver fibrosis stage was evaluated by transient elastography (TE) using a FibroScan^®^ device, and two groups were established for the analysis of liver stiffness (LS): mild and moderate stiffness (fibrosis F1 and F2; F1/2) and severe stiffness (fibrosis and cirrhosis F3 and F4; F3/4). Metabolic/cellular parameters were evaluated before and after antiviral treatment using standard methods: alkaline phosphatase (ALP), aspartate aminotransferase (AST), alanine aminotransferase (ALT), γ-glutamyl-transpeptidase (γ-GT), haptoglobin (Hp), total cholesterol (TC), high-density lipoprotein (HDL), low-density lipoprotein (LDL), triglycerides (TG), free iron (Fe), transferrin saturation (TS), total iron binding capacity (TIBC), ferritin (Ft), glycemia, insulin, Homeostatic Model Assessment for Insulin Resistance (HOMA-IR) and platelets count. The results were statistically analyzed using SPSS 24.0 for Windows. Results: Comparing the fibrosis stage before and after DAAs treatment, we verify a reduction in LS in 85.7% of patients and an improvement in liver fibrosis stage in 22.2% of them after DAAs treatment. Before DAAs treatment, patients showed a 2.410 risk for higher fibrosis stages (F3/4). Comparing metabolic/cellular parameters before and after DAAs treatment, patients showed lower ALP, AST, ALT, γGT, TG, Fe, TIBC, and Ft values and higher TC, LDL, and Hp values after treatment. As such, HCV elimination reduces iron overload and insulin resistance. On the other hand, it caused dyslipidemia, raising total cholesterol and LDL to levels outside the reference values. The improvement in the liver fibrosis stage by TE was mainly associated with higher baseline platelet count and HDL values and lower insulin resistance. Conclusions: With this study, we were able to contribute to the knowledge of the effects of HCV elimination with DAAs on liver disease and metabolic profile to improve the quality of treatment and follow-up of these patients after HCV elimination.

## 1. Introduction

The World Health Organization (WHO) estimates that about 71 million (1.1% of the world population) people have chronic hepatitis C infection.

Chronic hepatitis C (CHC) is not a single disease. It is a clinical and pathological syndrome with several causes and is characterized by different degrees of necrosis and hepatocellular inflammation [1].

This disease is a significant cause of liver transplantation and liver-related death worldwide [2]. About 60 to 85% of people infected with HCV develop a chronic stage of hepatitis C, with a 15 to 30% risk of developing liver cirrhosis or hepatocellular carcinoma (HCC) over 20 years. In patients with cirrhosis, the annual risk for HCC is 1–3% and 3–6% for hepatic decompensation. After hepatic decompensation, the risk of death in the following year is 15 to 20% [3,4]. HCC is known to be a leading cause of liver-related death and is the third most common cause of cancer death worldwide [5].

Hepatic manifestations of CHC are typically characterized by slowly progressive liver fibrosis, with progression from stage 0 (without fibrosis) to stage 4 (cirrhosis) occurring in approximately 0.10–0.15 units of fibrosis (median) per decade [6]. Fibrosis is a non-specific and often disproportionate response to tissue damage [7].

It is known that the outcome of CHC, namely liver fibrosis and its complications, is influenced by host factors (e.g., age, gender, obesity, steatosis, and genetics), viral factors (genotype and viremia) and environmental factors (e.g., alcohol consumption) [5,8].

HCV infection is a systemic disorder. Seventy-four percent of HCV patients have extrahepatic manifestations with different degrees of severity. These events cover a broad spectrum of various organs and systems, leading to extrahepatic complications (cardiovascular diseases, renal insufficiency, diabetes mellitus, insulin resistance, chronic systemic inflammation, oxidative stress, iron overload, dyslipidemia, etc.) [9,10,11,12,13,14,15,16]. Although liver disease is the leading cause of mortality in HCV-infected patients, they also show a higher mortality rate due to extrahepatic complications [10,14].

When HCV manages to evade the immune response surveillance, it will infect hepatocytes, thus causing an increase in oxidative stress and inducing the recruitment of inflammatory cells. The activation of myofibroblasts follows this and, consequently, an accumulation of collagen. In addition, the virus proteins will stimulate the secretion of profibrotic and proinflammatory cytokines in hepatocytes, which will directly induce activation and fibrogenesis in stellate cells [17].

Thus, liver fibrosis results in chronic liver damage and the accumulation of extracellular matrix proteins [18]. It is known that fibrogenesis in the liver is an active healing process of hepatic lesions and is reversible [18,19].

The fibrosis pathophysiology involves chronic liver damage with necroinflammation and requires the interaction of several types of liver cells and cytokines [20]. Liver damage causes HSCs to become more active, which triggers an increase in extracellular matrix synthesis [21]. The final consequences are liver dysfunction, portal hypertension, and increased risk for hepatocellular carcinoma (HCC) [22].

According to the EASL guidelines of noninvasive markers for the evaluation of fibrosis stage and more recently as recommended by Baveno VII, Transient Elastography (Fibroscan) is the noninvasive method for evaluating fibrosis stage [23].

The gold standard for treating HCV infection implies using direct-acting antivirals (DAAs). They act directly on the virus replicative cycle, inhibiting the non-structural proteins associated with viral replication.

Recommended treatment for adults is pangenotypic regimens for 8 to 12 weeks. DAA regimens show more than 95% cure rates, minimal side effects, and shortened therapy courses [24,25]. Undetectable HCV-RNA 24 weeks after treatment completion represents sustained virologic response (SVR) and virologic cure. An SVR is generally associated with an improvement in liver function, liver necroinflammation, and, consequently, an improvement in fibrosis.

The effects of HCV elimination with DAAs on the natural history of liver disease are not apparent in the literature.

A study from 2018 showed that viral eradication with DAAs reduces liver stiffness and steatosis in patients with CHC who received direct-acting antiviral therapy [26]. The same was found in a study from 2020 where 40 paired liver biopsies of CHC patients were analyzed before and after DAAs therapy and demonstrated a significant improvement in liver inflammation and fibrosis after eradication of HCV [27]. A large prospective study with more than 2000 patients with liver fibrosis followed up for at least one year after treatment revealed that in CHC, HCV clearance with DAAs is associated with the reversal of cirrhosis and the regression of hepatic fibrosis in about 50% of patients [28]. More recently, in 2022, a study with more than 1000 CHC patients showed an association between treatment with DAAs and regression of liver fibrosis from F3/4 to F1/2 in about 28% of patients [29].

Haptoglobin (Hp) is an acute-phase protein that can bind to circulating hemoglobin. It also acts as an antioxidant agent and has antibacterial activity. When there is an inflammatory stimulus, an increase in Hp plasma concentration occurs either locally (vascular) or systemically (extravascular) [30].

In humans, the induction of haptoglobin gene expression is mediated by interleukin-6 (IL-6). Three IL-6 responsive regulatory regions, A (157), B (−111), and C (−61), are present in the promoter of the *Hp* gene. These three regions constitute the binding site for hormones released during the acute phase response. The binding of hormones and cytokines to the regulatory regions will promote *Hp* gene transcription and increase Hp protein synthesis [30].

In the initial phase of an acute inflammatory response, Hp plays an active role, being a ligand for macrophage-1 antigen (Mac-1). Hp is involved in the early phase of neutrophil recruitment and can decrease neutrophil activity. It plays an immunomodulatory role in minimizing cell damage caused by the inflammatory response. Hp promotes neutrophil apoptosis as monocytes and macrophages are attracted to the injury site. Hp becomes more expressed throughout the cell repair process, increasing its activities and inhibiting gelatin deposition and fibroblast migration for wound healing and repair [30].

It is known that CHC patients also have signs of mild hemolysis, as indicated by significantly higher heme levels and lower Hp. Serum levels of this acute phase protein were shown to be lower in individuals with more severe liver disease and to be increased after DAAs therapy [31].

Platelets play a crucial role in hemostasis at the beginning of the coagulation cascade in response to vascular injury. Platelets will bind to the extracellular matrix to form a blood clot and will be activated upon exposure to collagen. Platelets have a range of functions other than coagulation, as they can synthesize many proteins [32].

When there are hepatic lesions, platelets are actively recruited to the liver and play a vital role in tissue regeneration, promoting the proliferation of hepatocytes [32].

Regarding the changes in platelet count after HCV clearance with DAAs, Badawi R. et al., in a study from 2021, showed an improvement in this parameter after a sustained virological response to DAAs [33].

HCV infection induces the accumulation of cytosolic lipid droplets (cLDs) in hepatocytes and the remodeling of the membrane by changing its lipid components. It provides structures and support for the efficient replication and morphogenesis of HCV. Similarly, HCV infection induces the upregulation of long fatty acyl chains in triglycerides (TG) and phosphatidylcholines (PC), and higher levels of polyunsaturated fatty acids (PUFAs) have also been reported upon HCV infection in cultured hepatocytes [34].

Lipids have a pivotal role in every step of the HCV viral life cycle (entry, replication, and assembly) and its circulation with the formation of complex lipoviral particles (LVP) [35,36]. These include Niemann-Pick C1-like 1 (NPC1L1), a receptor for cholesterol resorption, and scavenger receptor class B member 1 (SRB1), which acts to promote cholesterol uptake from lipoproteins and interacts with HCV envelope glycoprotein E2 to promote HCV entry. Another mechanism for HCV entry is mediated by hepatocyte very-low-density lipoprotein (VLDL) receptor, involving HCV envelope glycoprotein E2 and apoE. HCV may also interact with host cholesterol synthesis within hepatocytes. The secretion of HCV from hepatocytes involves complexing with apoE-containing host lipoproteins in the form of VLDL or HDL [37].

Much evidence points to the profound impact of HCV infection on lipid metabolism, even in the presence of other comorbidities like type II diabetes mellitus (T2DM) [38].

It is well known that HCV-infected patients have their lipid profile changed, and that is due to the interaction between HCV and host cholesterol synthesis pathways [39].

Previous studies showed that an increase in LDL after DAAs was related to carotid intima-media thickening and cardio-cerebral events in a relatively short follow-up of 26 months [40,41].

A more recent study reported a pro-atherogenic lipid pattern in patients infected with HCV during DAA treatment and in a short time [42,43]. Excessive accumulation of triglycerides within hepatocytes may lead to hepatic steatosis, and HCV is one of the causes. Steatosis can be a relevant factor for the aggravation of liver disease in CHC, leading to fibrosis and progressing to cirrhosis and hepatocellular carcinoma [44]. Excess lipids within hepatocytes or defective fatty acid oxidation are associated with increased lipotoxicity, which directly contributes to the development of fibrosis [45].

Iron is a physiologically essential nutrient for humans and vital for cellular homeostasis as it functions critically in many cellular processes [46,47].

However, iron can be biochemically dangerous because, in excess, it will damage tissues by promoting the synthesis of toxic reactive oxygen species to cell membranes, proteins, lipids, and DNA [47,48]. Under iron overload conditions, the liver is the primary receptor organ for this metal. High liver iron concentrations can result in hepatocellular lesions, fibrosis, and cirrhosis [49].

All the steps necessary to maintain homeostasis are regulated at a systemic and cellular level [50].

The liver is the main iron storage organ. It plays an essential role in iron metabolism, as it is here that transferrin (Tf), its main carrier protein, and ferritin (Ft), the primary storage protein, are synthesized [44].

CHC is associated with iron overload in 10–42% of individuals. In addition, 20–35% of patients with CHC have increased levels of transferrin saturation (TS), serum iron (Fe), and serum Ft, and these parameters correlate significantly and directly with liver fibrosis [46,51].

In chronic viral hepatitis, iron is believed to accumulate mainly due to an injury process in which infected hepatocytes release hemosiderin that is taken up by Kupffer cells (CKs). However, other mechanisms may also be implicated, such as hepatocyte regeneration, cytokine release, and changes in iron uptake due to chronic necroinflammation and intrahepatic shunting [52].

Inflammatory conditions are often associated with significant changes in systemic iron metabolism. The main factor responsible for these changes is the increased expression of hepcidin, which controls intestinal iron absorption and release from macrophages [53,54].

Moreover, HCV itself is known to influence iron absorption. This influence occurs through the oxidative stress-mediated negative regulation of hepcidin hormone expression [46].

HCV clearance with DAAs normalizes serum iron metabolism parameters, which occurs soon after antiviral treatment with a durable effect. Abnormalities of iron metabolism in CHC are either a direct effect of the virus or a consequence of viral infection, such as inflammation, that resolves with HCV elimination [55].

The liver is also responsible for maintaining glucose metabolism, storing glucose, and producing endogenous glucose from glycogen stores in the liver or gluconeogenesis. These activities contribute to preserving normal blood glucose levels [56].

HCV alters glucose metabolism by inducing inflammatory cascades and promoting insulin resistance.

Glucose transport to hepatocytes is conducted by glucose transporter 2 (GLUT2), downregulated by the HCV core protein. On the other hand, HCV infection promotes the overproduction of tumor necrosis factor-alpha (TNF-α) and other cytokines, such as IL-6, that inhibit insulin receptor substrate (IRS) and phosphatidylinositol three kinase (PI3K). This alteration in intracellular insulin signaling could block the activation of GLUT4 and reduce cells’ glucose uptake [57].

Higher HCV RNA levels were associated with higher Homeostatic Model Assessment for Insulin Resistance (HOMA-IR), and its improvement was correlated with a decrease in viral load [58,59,60]. Moreover, regarding the changes in glucose metabolism after DAAs therapy, a study showed that clearance of HCV with antiviral treatment resulted in the restoration of insulin sensitivity [61].

In the presence of liver damage, less insulin is absorbed by the hepatocyte and degraded, causing a situation of chronic hyperinsulinemia. Advanced fibrosis and liver cirrhosis may be accompanied by insulin resistance, causing liver inefficiency in metabolizing excess glucose. As a result, blood glucose levels increase.

Insulin resistance, impaired glucose tolerance, and T2DM are frequent extrahepatic manifestations of HCV [62,63,64], and some studies have shown a positive correlation between T2DM and insulin resistance (IR) and liver fibrosis progression in patients with chronic HCV infection [63,65,66].

The primary objective of the study was to determine if, in patients with CHC, HCV clearance with DAAs changes the severity of liver disease (liver stiffness and liver fibrosis stage by TE) and the metabolic/cellular profile (liver damage, acute inflammatory response, lipid, iron, and glucose metabolisms, and tissue regeneration).

The secondary objective was to identify baseline metabolic/cellular parameters that could predict the improvement in liver fibrosis after DAA treatment.

## 2. Materials and Methods

*Populations*: A group of 329 patients with CHC were prospectively studied. From these, 134 were also evaluated after being treated with Sofosbuvir/Velpatasvir 1 pill per day for 12 weeks, a pangenotypic DAA. Table 1 resumes the main characteristics of the studied populations. The two columns before treatment refer to all populations (*n* = 329) and those treated (*n* = 134). We can observe that they are mainly male and have fibrosis stages F1/2. They have a borderline average BMI value and are around 50 years old.

Patients were selected, examined, adequately informed, and consented following the WMA Helsinki Declaration [67].

Inclusion criteria (before treatment): presence of positive RNA and anti-HCV for more than six months; age 18 years or older and informed consent.

Inclusion criteria (after treatment): sustained response (HCV-RNA undetec 3 and 6 months after viral load 0 UI/mL according to European Association for the Study of Liver (EASL) guidelines; age 18 years or older and informed consent.

Exclusion criteria: other chronic liver diseases (viral hepatitis A and/or B, autoimmune diseases, and other genetic and/or metabolic diseases); concurrent infection with HIV; alcohol consumption > 40 gr/day; pregnant women and individuals with impaired intellectual capacity.

*Liver fibrosis evaluation*: Liver stiffness (LS) was evaluated by transient elastography (TE) using a FibroScan^®^ device (Echosens, Paris, France) with a 5-MHz ultrasound transducer mounted on the axis of a vibrator. The vibrator generates a painless vibration (frequency 50 Hz and amplitude 2 mm) like a “flick”, generating a shear wave propagating through the skin and the subcutaneous tissue into the liver. The velocity of the wave is directly related to the LS. The median value of 10 successful acquisitions was expressed in kilopascals (kPa), with a success rate of at least 60% and an interquartile range (IQR) lower than 30%. LS cut-off values validated in the Gastroenterology and Hepatology Department, Hospital de Santa Maria, Lisbon, Portugal (liver biopsy analysis of 110 patients, Scheuer classification) were used to establish fibrosis stages: 5.43 kPa for F ≥ 2 (PPV 0.78; NPV 0.67); 8.18 kPa for F ≥ 3 (PPV 0.95 NPV 0.93); 12 kPa for F = 4 (PPV 0.93; NPV 0.93). The average value was ≤4.9 kPa [68].

*HCV-RNA*: Serum HCV-RNA was evaluated by Real-Time PCR Taqman from Roche Diagnostics (test sensitivity < 15 IU/mL).

*Metabolic/cellular evaluation*: Metabolic/cellular parameters were evaluated before and after antiviral treatment using standard methods from the Clinic Pathology Department of the hospital (reference values described).

Liver damage: alkaline phosphatase (ALP ≤ 129 UI/L), aspartate aminotransferase (AST ≤ 34 IU/l), alanine aminotransferase (ALT ≤ 49 IU/l), and γ-glutamyl-transpeptidase (γ-GT < 38 IU/l) Acute inflammatory response: haptoglobin (Hp ≤ 200 mg/dL); Lipid metabolism: total cholesterol (TC ≤ 4.92 mmol/L), high-density lipoprotein (HDL ≥ 1.04 mmol/L), low-density lipoprotein (LDL ≤ 2.8 mmol/L) and triglycerides, (TG ≤ 1.69 mmol/L); Iron metabolism: free iron (Fe ≤ 170 μg/dL), transferrin saturation (TS ≤ 40%), total iron binding capacity (TIBC ≤ 450 μg/dL), and ferritin (Ft ≤ 291 ng/mL); Glucose metabolism: glycemia (glycemia ≤ 110 mg/dL), insulin (insulin ≤ 25 mcU/mL), and Homeostatic Model Assessment for Insulin Resistance (HOMA-IR) ≤ 2); Tissue regeneration: platelets count (≥150,000/µL).

*Statistical analysis*: Statistical analysis was performed using SPSS 24.0 for Windows. Data were inserted into a database built in this same program, safeguarding the confidentiality of the participants’ identities.

Metabolic parameters were treated as continuous variables.

Two groups were established for the analysis of liver fibrosis. Patients with mild and moderate fibrosis (F1 and F2; F1/2) and patients with severe fibrosis and cirrhosis (F3 and F4; F3/4) were grouped so that the frequencies would not be too low in some cases.

Descriptive analysis was performed assuming univariate and bivariate analysis. The non-parametric Kolmogorov–Smirnov test first tested the normality for continuous variables. As there was at least one variable with a non-normal distribution, all variables were analyzed using non-parametric tests and were described as mean and 95% confidence interval. Absolute and relative frequencies were used to define the categorical variables. In the bivariate analysis, categorical variables were compared using the Chi-square test or Fisher’s exact test, and the Odds Ratio (OR) was calculated with their respective confidence intervals whenever justifiable. Statistical comparisons were performed for continuous variables using the non-parametric Mann–Whitney and Kruskal–Wallis tests. We use Paired Sample Tests to make comparisons before and after DAA treatment. Whenever the compared groups differed in gender, age, or BMI, statistical analysis was conducted with adjustments for the parameter/s that differed. It was considered a statistically significant result for a *p*-value < 0.05.

## 3. Results

### 3.1. Fibrosis Stage and Metabolic/Cellular Parameters before and after DAAs Treatment

Comparing the fibrosis stage before and after DAAs treatment we found a significantly increased frequency of F1/2 patients after DAAs treatment (almost 20%). We also verified a reduction in LS in 85.7% of patients and an improvement in liver fibrosis stage in 22.2% of them after DAAs treatment.

Before DAAs treatment, patients showed a 2.410 risk for higher fibrosis stages (F3/4) (Table 2).

As we do not have a proportionate distribution of cases within F1/2 and F3/4 pools, as seen in Table 1, the same comparison was made using all the fibrosis stages separately (F1, F2, F3, and F4). We obtained statistically significant results (*p* < 0.001) with a decrease in stages F3 and F4 after treatment (F3 before = 9.7% vs. F3 after = 8.2%; F4 before = 29.1% vs. F4 after = 14.2%).

Comparing metabolic/cellular parameters before and after DAAs treatment, patients showed lower ALP, AST, ALT, γGT, TG, Fe, TIBC, and Ft values and higher TC, LDL, and Hp values after treatment. HDL, TS, Platelets, Glycaemia, Insulin, and HOMA-IR did not change significantly before and after treatment (Table 3).

### 3.2. Association of Metabolic/Cellular Parameters with Fibrosis Stage before DAAs Treatment

Baseline metabolic/cellular parameters were compared between patients with higher fibrosis stages (F3/4) and those with lower ones (F1/2) before DAAs treatment. Patients with severe fibrosis showed higher ALP, AST, ALT, γGT, Fe, TIBC, Ft, glycemia, and HOMA-IR values and lower Hp, TC, LDL, and platelet count values. HDL, TG, and TS did not show significant results (Table 4).

### 3.3. Association of Baseline Metabolic/Cellular Parameters with the Improvement in Fibrosis Stage after DAAs Treatment

Within DAAs-treated patients, 18.8% showed an improvement in liver fibrosis as they passed from higher fibrosis stages (F3/4) to lower fibrosis ones (F1/F2). On the other hand, 79.0% maintained their initial fibrosis stage (58.9% F1/2 and 20.1% F3/F4). Only 2.2% showed a worsening of the fibrosis stage (F1/2 to F3/4).

Comparing baseline metabolic/cellular parameters of those who improved liver fibrosis with those who did not, we found increased values of HDL and platelet counts and decreased values of insulin and HOMA-IR for the first ones (Table 5). So, these changes were associated with improved liver fibrosis despite the overall assessment of these parameters comparing before and after treatment (Table 3) and F1/2 with F3/4 before treatment (Table 4) revealed no significant changes.

Regarding only patients with cirrhosis (F4), 51.3% regressed the fibrosis stage to mild, moderate, or severe (less than F4), while 48.7% stayed cirrhotic.

Comparing baseline biochemical parameters between these two groups and applying the same statistic tests as before (Table 5), we obtained the following results: those who improved liver fibrosis compared with those who stayed cirrhotic showed increased platelets count and decreased values of glycemia, insulin, and HOMA-IR.

## 4. Discussion

Answering the primary objective, HCV clearance with DAAs improves liver fibrosis as we found a significantly increased frequency of F1/2 patients after DAAs treatment (almost 20%).

Comparing the LS and fibrosis stages before and after DAAs treatment, we verified a reduction in LS in 85.7% of patients and an improvement in liver fibrosis stage in 22.2% of them after DAAs treatment. The initial process of fibrogenesis in viral hepatitis is associated with the degree of necroinflammation. During this process, type III collagen fibers accumulate. The elimination of inflammation induced by antiviral therapy is associated with a reduction in type III collagen fibers [69]. This explains the decrease in elastography values at an early stage in the cure of the infection, even if not accompanied by a reduction in the liver fibrosis stage.

The issue regarding using different LS cut-off values to classify the patients according to liver fibrosis stage after HCV clearance with DAAs is under discussion. However, it is agreed that performing a liver biopsy in patients to assess the liver fibrosis stage after SVR is not ethical. There are no LS cut-off values to evaluate liver fibrosis after antiviral therapy with DAAs. The international guidelines continue to recommend the use of TE to follow-up patients after SVR [70,71]. A 2021 study showed that even though TE accuracy is lower three years after HCV clearance compared to pre-therapy, it remains the only variable associated with cirrhosis after HCV elimination [72].

In addition, HCV clearance also changes metabolic/cellular parameters of liver injury—normalization of ALP, AST, ALT, and γGT; acute inflammatory response—higher values of Hp; lipid metabolism—lower values of TG and higher values of TC and LDL; iron metabolism—lower values of Fe, TIBC, and Ft.

Regarding the secondary objectives, we found that higher fibrosis stages (F3/4) before DAAs treatment were associated with higher baseline values of ALP, AST, ALT, γGT, Fe, TIBC, Ft, glycemia, and HOMA-IR and lower values of Hp, TC, LDL, and platelets count.

The improvement in liver fibrosis (F3/4 to F1/2) after DAAs treatment was associated with higher baseline values of HDL and platelet count and lower baseline values of insulin and HOMA-IR, despite the overall assessment of these parameters comparing before and after treatment and comparing F1/2 with F3/4 before treatment revealing no significant changes.

Liver disease caused by HCV infection translates into structural changes in the liver that result in several stages of liver fibrosis and serum non-invasive biomarkers of liver damage, necroinflammation, and fibrosis [73].

As expected, in our study, higher ALP, AST, ALT, and γGT values were associated with more advanced fibrosis stages before and after DAAs treatment. These results agree with a lot of other studies since the association between these enzymes and liver injury is well established [8,51,73,74,75,76,77]. As expected, our study also revealed decreased values of liver damage parameters after DAAs therapy. These results agree with other studies that report a normalization of these biomarkers after HCV elimination [78,79,80].

Concerning Hp, we found lower values for patients with higher fibrosis stages (F3/4). This result is supported by the studies of Myers et al. and Grigorescu et al. where a negative association was found between Hp and fibrosis stages in HCV patients. The decreased Hp values may be explained by the increased levels of hepatocyte growth factor, which stimulates a decline in Hp synthesis [81,82,83].

In our study, Hp increased after the elimination of HCV with DAAs, probably reflecting a process of cellular healing and repair [30].

HCV clearance was accompanied by increased TC and LDL and decreased TG. Levels of HD showed no significant changes.

There is clear evidence that HCV eradication produces a simultaneous increase in TC and LDL levels, creating a combination of circumstances that might aggravate the risk of atherosclerosis and cardiovascular disease. Regarding HDL, the results of the different studies are contradictory since some show an increase in HDL levels, others a decrease, and others, no significant differences [84,85,86,87]. In the case of TG, contradictory results have also been reported, with minimal or absent changes [87,88], or a decrease [89,90], as we found in our study.

A study from 2022 reported that TC and LDL showed the earliest and most pronounced increase after DAAs treatment, and this result was aligned with most studies, including retrospectives, with short follow-ups, or with heterogeneous populations. On the other hand, TG and HDL showed less abrupt and more gradual increases [42]. These results aligned with those observed by Inoue et al., Gonzalez-Colominas et al., and Shimizu et al., all long follow-up studies [87,88,91]. Interestingly, studies that reported no differences in HDL and TG, such as those from Cheng et al., Jain et al., and Ichikawa et al., had reduced follow-ups [92,93,94].

Our study has a short follow-up of six months, which may lead to non-significant changes in HDL levels.

Higher fibrosis stages before treatment were associated with lower values of TC and LDL. This is what we expected as the liver plays an essential role in lipid metabolism, e.g., synthesis and transportation. Liver injury reduces lipoprotein biosynthesis, resulting in an abnormal lipid profile in those with severe liver dysfunction [95].

After treatment, higher fibrosis stages were associated with lower baseline values of HDL. Moreover, in our study, the improvement in liver fibrosis after HCV elimination was associated with higher baseline levels of HDL. These results account for HDL’s protective role for higher fibrosis stages after HCV clearance. HDL is thought to be an essential endogenous inhibitor of inflammatory responses [96]. Chronic inflammation in the liver can lead to the activation of fibrogenic pathways, leading to fibrosis, which can develop into cirrhosis, hepatocellular carcinoma, and death [97]. Previous studies reported that lipopolysaccharide (LPS)-induced overproduction of pro-inflammatory cytokines by monocytes could be abolished by incubation of whole blood with reconstituted HDL [98,99].

For Fe, Ft, and TIBC, we observed higher values of these parameters associated with higher stages of liver fibrosis before DAAs treatment which is partially in agreement with the studies of Guyader et al. and Vagu et al. [51,77]. In a situation of liver inflammation, iron may be released from damaged hepatocytes [77]. Some studies suggest that high iron levels may aggravate hepatic necroinflammatory activity in CHC and accelerate the progression of liver fibrosis [100,101]. Moreover, iron accumulation in CHC causes oxidative stress and consequent liver damage, which increases the necrosis/apoptosis of hepatocytes, the activation of hepatic stellate cells, and fibrogenesis through the proliferation of actin and collagen [65].

Regarding Ft, we obtained the same results as Vagu et al.; serum Fe levels are essential in determining the severity of liver disease associated with liver fibrosis and necroinflammatory activity. As with Fe, Ft is released by damaged hepatocytes when it is in an inflammatory situation, and the higher the inflammatory process, the more Fe is released [77]. The same study reported lower levels of TIBC associated with more severe fibrosis stages. This result contradicts the result obtained in our study, as we noted that for higher degrees of fibrosis, the mean TIBC values are also higher. We did not find studies that corroborate our findings.

Regarding the effects of HCV elimination in the parameters of iron metabolism, we observed a decrease in the above ones (Fe, Ft, and TIBC) after DAAs treatment.

In the presence of HCV infection, hepcidin transcription is suppressed, causing a decrease in its expression, which leads to increased expression of ferroportin in enterocytes and reticuloendothelial macrophages, thus resulting in increased duodenal iron transport and iron release from macrophages [101]. Moreover, the increase in hepatic iron in viral hepatitis may be due to a defensive process adopted during infections, which will involve iron accumulation by liver cells to limit the access of pathogens and thus inhibit their proliferation [102]. With HCV elimination, these two processes become the opposite, leading to a decrease in the values of iron parameters. This result is in concordance with the study from Hasan Y. et al. that reported the normalization of serum iron parameters after HCV clearance with DAAs soon after antiviral treatment, suggesting a direct effect of the virus on iron metabolism. On the other hand, this study also showed that this normalization has a durable impact, meaning that the abnormalities of iron metabolism in CHC may also result from a viral infection, such as inflammation, that resolves with HCV elimination [55].

Concerning platelet count, the results obtained in the studies conducted by Murawaki et al. and Lima et al. corroborate the results obtained in our study, as we found low platelet count is associated with high stages of fibrosis [103,104].

According to Fusegawa H., et al. this phenomenon occurs since thrombocytopenia (reduction in the number of platelets in the blood) is a common complication of chronic hepatopathies in advanced stages [105]. The inflammatory process induced by HCV leads to the consumption of platelets by the spleen [106]. Moreover, reduced platelet count may directly result from liver damage as the production of thrombopoietin is compromised [107].

Another interesting result is that a higher baseline number of platelets was present in patients who improved liver fibrosis after HCV clearance, emphasizing the importance of this blood component in liver regeneration.

When parameters of glucose metabolism were studied, we found that higher stages of fibrosis were associated with higher glycemia, insulin, and HOMA-IR values before and after treatment and an improvement in liver fibrosis in patients with lower baseline values of insulin and HOMA-IR.

These were the expected results, as damaged hepatocytes degrade less insulin, increasing its values. Moreover, insulin resistance and higher glucose values may accompany a more advanced liver disease (fibrosis) [62].

Other studies showed a positive correlation between T2DM and IR and liver fibrosis progression in patients with chronic HCV infection [14,65,66].

Although we did not observe any change in glucose metabolism parameters after DAAs treatment, a study from 2022 showed a reduction in insulin upon DAAs treatments which translated to an improved homeostatic model assessment (HOMA-IR) [42].

## 5. Conclusions

Our study showed a global improvement in liver fibrosis by TE after HCV clearance with DAAs, as we found a significantly increased frequency of F1/2 patients after DAAs treatment (almost 20%).

When we considered the metabolic/cellular parameters before antiviral treatment, we concluded that the improvement of liver fibrosis from F3/4 (severe or cirrhosis) to F1/2 (mild or moderate) or even the regression of cirrhosis (F4 to F1/2/3) was mainly associated with higher baseline values of platelets count, and HDL and less IR. In that way, monitoring these parameters before starting antiviral therapy can help assess the prognosis of liver disease after treatment.

In our study, HCV elimination reduced iron overload and IR. On the other hand, it causes dyslipidemia, raising TC and LDL to levels outside the reference values. It seems relevant to follow up with the patients even after HCV eradication to understand whether these increases are transient and the immediate result of HCV elimination or if they persist and can increase the risk of cardiovascular disease, especially in patients with other comorbidities.

Despite the effective elimination of HCV, which is extremely relevant as it eradicates the main factor causing the disease, it seemed pertinent to understand to what extent HCV clearance eliminates or attenuates all the systemic alterations already induced by the virus during infection and chronicity.

Although this study allows us to define an association but not a cause–effect relationship between DAAs and the reduction in liver fibrosis stage, we were able to contribute to the knowledge of the effects of HCV elimination with DAAs on liver disease and metabolic profile to improve the quality of treatment and a need of a long-term follow-up for patients with advanced chronic liver disease, even after HCV elimination.

## Figures and Tables

**Table 1:** Characterization of CHC patients before and after DAAs treatment.

Parameter	Before DAAs Treatment (*n* = 329)		Before DAAs Treatment (*n* = 134)		After DAAs Treatment (*n* = 134)	
	Mean	95% CI	Mean	95% CI	Mean	95% CI
Age (years)	48.93	[47.57–50.28]	53.42	[51.47–55.36]	53.42	[51.47–55.36]
BMI (Kg/m^2^)	25.25	[24.80–25.06]	25.06	[20.78–26.10]	25.06	[20.78–26.10]
HCV-RNA (IU/mL)	2.16 × 10^6^	[1.51 × 10^6^–2.82 × 10^6^]	2.16 × 10^6^	[2.03 × 10^6^–4.8 × 10^6^]	0.00	-
Parameter	N	%	N	%	N	%
Gender						
Female	124	37.7	58	43.3	58	43.3
Male	205	62.3	76	56.7	76	56.7
Liver fibrosis						
F_1_	84	25.6	29	21.6	59	44.0
F_2_	130	39.5	53	39.6	45	33.6
F_3_	35	10.6	13	9.7	11	8.2
F_4_	80	24.3	39	29.1	19	14.2

95% CI—95% confidence interval for mean; n—number of patients; %—percentage of patients.

**Table 2:** Fibrosis stage before and after DAAs treatment.

	FIBROSIS STAGE (LS)		*p*-Value *	OR	95% CI
	F1/2	F3/4			
Before DAAs treatment; n (%)	82 (61.2)	52 (38.8)	0.001	2.410	[1.416–4.100]
After DAAs treatment; n (%)	104 (77.6)	30 (22.4)		1	

* Fisher exact test; OR—odds ratio; 95% CI—95% confidence interval.

**Table 3:** Metabolic/cellular parameters before and after DAAs treatment.

Parameter	Before DAAs Treatment		After DAAs Treatment		*p*-Value *
	Mean	95% CI	Mean	95% CI	
ALP (UI/L)	80.37	[74.65–86.10]	71.70	[67.48–75.92]	<0.001
AST (UI/L)	57.70	[51.36–64.04]	24.59	[22.52–26.67]	<0.001
ALT (UI/L)	76.02	[66.48–85.55]	23.38	[20.70–26.06]	<0.001
γGT (UI/L)	88.27	[72.68–103.85]	30.62	[23.10–38.14]	<0.001
Hp (mg/dL)	101.54	[94.72–108.36]	115.88	[106.13–125.62]	<0.001
TC (mmol/L)	4.42	[4.31–4.54]	5.13	[4.95–5.30]	<0.001
HDL (mmol/L)	1.50	[1.35–1.65]	1.71	[1.57–1.86]	0.541
LDL (mmol/L)	2.47	[2.35–2.58]	3.13	[2.98–3.28]	<0.001
TG (mmol/L)	1.24	[1.16–1.32]	1.05	[0.97–1.13]	0.017
Fe (μg/dL)	123.16	[116.77–129.54]	111.07	[103.61–118.53]	0.003
TS (%)	40.39	[37.02–43.77]	37.14	[34.03–40.25]	0.104
TIBC (μg/dL)	320.53	[311.76–329.31]	309.57	[298.86–320.29]	0.013
Ft (ng/mL)	244.66	[211.52–277.81]	148.80	[128.03–169.58]	<0.001
Platelets count (U/µL)	2.05 × 10^5^	[1.92 × 10^5^–2.17 × 10^5^]	2.04 × 10^5^	[1.89 × 10^5^–2.18 × 10^5^]	0.456
Glycaemia (mg/dL)	94.62	[90.91–98.33]	96.52	[91.67–101−38]	0.575
Insulin (mcU/mL)	15.76	[12.94–18.59]	12.35	[10.97–13.72]	0.123
HOMA-IR	3.85	[3.08–4.61]	3.08	[2.68–3.47]	0.168

* Paired Sample Test; 95% CI—95% confidence interval for mean.

**Table 4:** Association of baseline metabolic/cellular parameters with fibrosis stage before DAAs treatment.

Baseline Parameter	F_1/2 (before DAAs Treatment)_		F_3/4 (before DAAs Treatment)_		*p*-Value *
	Mean	95% CI	Mean	95% CI	
ALP (UI/L)	72.06	[68.60–75.51]	93.15	[85.03–101.28]	<0.001
AST (UI/L)	49.67	[45.48–53.86]	88.26	[77.27–99.26]	<0.001
ALT (UI/L)	79.91	[70.34–89.48]	116.07	[98.79–133.36]	<0.001
γGT (UI/L)	55.26	[48.86–61.66]	118.53	[95.74–141.33]	<0.001
Hp (mg/dL)	110.68	[102.62–118−75]	86.39	[74.79–97.99]	<0.001
TC (mmol/L)	4.52	[4.37–4.67]	4.24	[4.05–4.43]	0.005
HDL (mmol/L)	1.58	[1.35–1.80]	1.37	[1.24–1.50]	0.091
LDL (mmol/L)	2.58	[2.43–2.71]	2.29	[2.10–2.47]	0.006
TG (mmol/L)	1.22	[1.11–1.34]	1.27	[1.15–1.39]	0.084
Fe (μg/dL)	115.42	[108.47–122.38]	135.67	[123.56–147.78]	0.006
TS (%)	39.56	[34.72–44.40]	41.66	[37.27–46.04]	0.258
TIBC (μg/dL)	310.79	[300.40–321.17]	335.31	[320.09–350.54]	0.004
Ft (ng/mL)	189.89	[167.58–212.19]	336.98	[259.04–414.92]	0.002
Platelets count (U/µL)	2.22 × 10^5^	[2.13 × 10^5^–2.31 × 10^5^]	1.75 x10^5^	[1.61 × 10^5^–1.90 × 10^5^]	<0.001
Glycaemia (mg/dL)	90.54	[87.14–93.94]	101.61	[93.48–109.73]	0.026
Insulin (mcU/mL)	15.93	[11.68–20.19]	15.47	[13.11–17.83]	0.060
HOMA-IR	3.70	[2.60–4.80]	4.12	[3.30–4.95]	0.010

* Mann-Whitney Test; 95% CI—95% confidence interval for mean.

**Table 5:** Comparison of baseline metabolic/cellular parameters between patients who improved liver fibrosis and those who did not.

Baseline Parameter	F_3/4 (before DAAs Treatment)_ to F_1/2 (after DAAs Treatment)_		F_3/4 (before DAAS Treatment)_ to F_3/4 (after DAAs Treatment)_		*p*-Value *
	Mean	95% CI	Mean	95% CI	
ALP (UI/L)	83.04	[71.10–94.98]	101.30	[81.13–121.47]	0.179
AST (UI/L)	79.96	[57.10–102.81]	84.85	[69.36–100.34]	0.234
ALT (UI/L)	101.79	[70.65–132.94]	99.56	[75.48–123.63]	0.692
γGT (UI/L)	134.63	[78.44–190.81]	126.37	[82.01–170.73]	0.727
Hp (mg/dL)	100.05	[76.57–123.52]	78.24	[59.70–96.78]	0.115
TC (mmol/L)	4.25	[3.84–4.65]	4.38	[4.00–4.76]	0.771
HDL (mmol/L)	1.71	[1.40–2.02]	1.44	[1.17–1.71]	0.039
LDL (mmol/L)	2.09	[1.78–2.39]	2.34	[2.05–2.63]	0.179
TG (mmol/L)	1.26	[1.06–1.46]	1.33	[1.11–1.55]	0.880
Fe (μg/dL)	128	[107.22–148−78]	132.19	[113.29–151.08]	0.514
TS (%)	36.96	[29.90–44.02]	40.52	[33.17–47.86]	0.325
TIBC (μg/dL)	329.65	[307.47–351.83]	343.15	[311.54–374.76]	0.381
FT (ng/mL)	277.26	[167.53–386.99]	331.78	[230.38–433.18]	0.465
Platelets count (U/µL)	2.11 × 10^5^	[1.78 × 10^5^–2.44 × 10^5^]	1.49 × 10^5^	[1.16 × 10^5^–1.82 × 10^5^]	0.003
Glycaemia (mg/dL)	95.87	[82.90–108.84]	113.04	[93.33–132.75]	0.070
Insulin (mcU/mL)	11.05	[8.53–13.56]	19.00	[14.20–23.80]	0.004
HOMA-IR	2.76	[1.74–3.79]	5.82	[3.98–7.66]	0.006

* Mann-Whitney Test; 95% CI—95% confidence interval for mean.

## Data Availability

The datasets used and analyzed during the current study are available from the corresponding author upon reasonable request.
